# Oncoplastic Approaches to Breast Conservation

**DOI:** 10.4061/2011/303879

**Published:** 2011-08-22

**Authors:** Dennis R. Holmes, Wesley Schooler, Robina Smith

**Affiliations:** ^1^Kenneth Norris Comprehensive Cancer Center and Division of Surgical Oncology, Keck School of Medicine, University of Southern California, 1441 Topping Tower, Suite 7415, Los Angeles, CA 90033, USA; ^2^USC Healthcare Consultation Center, Suite 514, HCC 514 M/C 9202, Los Angeles, CA 90089-9202, USA; ^3^21541 N. Harbor Boulevard, Suite 3100, Fullerton, CA 92835, USA

## Abstract

When a woman is diagnosed with breast cancer many aspects of her physical, emotional, and sexual wholeness are threatened. The quickly expanding field of oncoplastic breast surgery aims to enhance the physician commitment to restore the patient's image and self-assurance. By combining a multidisciplinary approach to diagnosis and treatment with oncoplastic surgery, successful results in the eyes of the patient and physician are significantly more likely to occur. As a way to aid oncoplastic teams in determining which approach is most suitable for their patient's tumor size, tumor location, body habitus, and desired cosmetic outcome we present a review of several oncoplastic surgical approaches. For resections located anywhere in the breast, the *radial ellipse segmentectomy incision* and *circumareolar approach for segmental resection* are discussed. For resections in the upper or central breast, *crescent mastopexy*, the *batwing incision*, the *hemibatwing incision*, *donut mastopexy*, *B-flap resection*, and the *central quadrantectomy* are reviewed. For lesions of the lower breast, the *triangle incision*, *inframammary incision*, and *reduction mastopexy* are discussed. Surgeons who are interested in adding oncoplastic breast conserving therapies to their skill sets are encouraged to implement these surgical techniques where applicable and to seek out breast fellowships or enhanced training when appropriate.

## 1. Introduction

The diagnosis of breast cancer is a life-changing experience. Not only does it bring the woman face to face with her mortality, but also surgical treatment of breast cancer is accompanied by physical changes to the breast and body that may significantly, and often permanently, alter her perception of her physical, emotional, and sexual wholeness. 

Since the Early Breast Cancer Trialists' Collaborative Group established the equivalency of mastectomy and breast conserving therapy in 1985, breast conserving surgery has remained the optimal surgical treatment for the breast cancer patient [1]. The goals of breast conserving surgery are the removal of breast cancer with an adequate surgical margin and maintenance of a breast that is cosmetically acceptable to the patient. Mastectomy with or without breast reconstruction is the treatment of choice when tumor resection and cosmesis is unattainable. Given the understandable desire to preserve a sense of wholeness, it is not surprising that many women consider mastectomy to be an unacceptable cosmetic alternative to breast conserving surgery.

Increasing use of mammographic screening and neoadjuvant chemotherapy has rendered 70–80% of breast cancer patients as potential candidates for breast conserving surgery (BCS). Nonetheless, BCS remains highly underutilized, with nearly 50% of women either selecting or being advised to undergo mastectomy [2]. Although underutilization of BCS may partly reflect limited access to radiotherapy or the patients' desire to minimize the risk of local recurrence, surgeon judgment is of paramount importance in assessing the potential for cosmesis. In addition, aesthetic success in BCS is dependent upon a variety of patient- and tumor-specific factors. Small breast size, ptotic breast shape, large body habitus, large tumor size, central, medial, or lower quadrant tumor location, segmental or multifocal tumor distribution, tumor re-excision, and resection of >20% breast volume have all been identified as predictors of poor cosmesis [3, 4]. 

The goal of optimizing the cosmetic and oncologic outcomes of BCS has been addressed in recent years by the emergence of the field of oncoplastic surgery. Originally defined as an assortment of volume replacement techniques performed by plastic surgeons to replace all or part of the resected breast volume with myocutaneous tissue flaps, the definition of oncoplastic surgery has more recently been expanded to include a wide range of volume displacement or volume redistribution procedures performed by breast surgeons and general surgeons to optimize breast shape and breast volume following breast cancer surgery [5–9]. Also included in the definition of “oncoplasty” is the surgical correction of breast asymmetry achieved by reducing or reconstructing the contralateral breast. The emergence of oncoplastic surgery reflects a growing appreciation for the importance of breast cosmesis and the willingness of many surgeons to obtain advanced training to improve cosmetic outcomes for their patients. Thus, the traditional emphasis on scar placement (i.e., Langer's lines and Kraissl's lines) and skin preservation is gradually being replaced or complimented by an appropriate emphasis on breast shape, volume, and symmetry. While traditional volume replacement oncoplastic procedures (e.g., myocutaneous flap reconstruction) remain beyond the skill set of most oncologic surgeons, a wide range of volume displacement procedures are relatively easy to learn and can be gradually incorporated into an oncologic surgery practice. 

## 2. Multidisciplinary Approach

Oncoplastic surgery requires a multidisciplinary approach to breast cancer care characterized by close collaboration between the breast surgeon, radiologist, radiation oncologist, and, when appropriate, plastic surgeon, medical oncologist, genetic counselor, and psychologist all working together to help the patient achieve the best possible surgical outcome [10]. The general requirement for adjuvant radiotherapy calls for coordination with the radiation oncologist. The timing of surgery should also be coordinated with the medical oncologist for patients receiving neoadjuvant chemotherapy or endocrine therapy. Genetic counseling and psychoanalysis should facilitate treatment planning (e.g., contralateral prophylactic mastectomy) and psychological well-being. Upon making a decision that breast conservation is achievable and desirable, the definite approach to management of the index and contralateral breast is selected by consensus between the surgeon, patient, and plastic surgeon. Accurate diagnostic evaluation and lesion localization should be planned with the radiologist.

## 3. Management of the Contralateral Breast

Breast asymmetry resulting from BCS can be managed with contralateral breast reduction or mastopexy to restore breast symmetry. Symmetrization surgery can be performed at the time of BCS, at a second operation, or may be indefinitely deferred, depending on the interests of the surgeon, the patient's wishes, the clinical setting, and the availability of plastic surgery expertise [11]. The timing of contralateral breast surgery is controversial owing to concerns about surgical margins and the potential need for re-excision or conversion to mastectomy, changes in breast volume following radiotherapy, and breast edema resulting from breast or axillary surgery.

## 4. Before Getting Started: General Principles

Since oncoplastic procedures may offer the patient her best or last chance of achieving cosmetic success, oncoplastic surgeons must accept a heightened responsibility to achieve oncologic success at the initial breast operation. Inadequate surgical margins not only compromise the oncologic outcome, but breast re-excision may diminish an initially aesthetic result, increase breast asymmetry, or necessitate conversion to mastectomy. To improve the odds of initial success, surgeons contemplating an oncoplastic approach should adhere to the following recommendations.

### 4.1. Restrict Oncoplastic Surgery to Definitive Care

Oncoplastic surgical procedures should be reserved for definitive therapeutic management of the diagnosed breast lesion. For undiagnosed patients, diagnostic biopsies should be obtained using minimally invasive breast biopsy techniques (e.g., core needle biopsy) to avoid extensive surgery in patients with benign breast conditions that may not require surgical excision. Limiting oncoplastic surgery to the therapeutic management of breast cancer also avoids removal of excess breast tissue or placement of surgical incisions that may jeopardize the perfusion of subsequent glandular or dermoglandular tissue flaps [12]. In the event that surgical excision is required for diagnostic purposes (e.g., for radial scar, atypical ductal hyperplasia, or papillary lesions), incision placement should anticipate the potential use of oncoplastic techniques in subsequent procedures.

### 4.2. Apply Radiopaque Markers to Surgical Margins

The cornerstone of oncoplastic breast conserving surgery is the mobilization and redistribution of the breast gland to reconstruct the breast mound. Since nearly all patients are expected to undergo adjuvant radiotherapy and infrequently re-excision, placement of multiple radiopaque tissue markers (e.g., Hemoclips) along the surgical margins should be performed to facilitate radiation planning, margin re-excision, and subsequent mammographic surveillance [13].

### 4.3. Use Multiple Bracketing Wires

Wire localization of nonpalpable or indistinct lesions using multiple bracketing wires is recommended to clearly define the lesion and the desired surgical margins. Placement of only a single wire through the center of the lesion should be avoided for larger lesions as it increases the probability of close or positive margins [14, 15]. Optimal wire localization should include placement of localizing wires on either side of a lesion (e.g., cranial and caudal or medial and lateral), but placement of an additional wire superficial to a lesion may also be used when preservation of the overlying skin is planned. Having the radiologist mark the skin overlying a nonpalpable lesion will also aid the surgeon in honing in on a lesion and reduces the need for excessive tunneling through the breast parenchyma.

### 4.4. Utilize Intraoperative Ultrasound for Sonographically Apparent Lesions

Intraoperative ultrasound is recommended for surgeons who are experienced in the use and interpretation of breast ultrasound [16, 17]. The value of intraoperative ultrasound for oncoplastic surgical resection is most apparent when approaching a lesion from the posterior aspect of the breast, such as in the inframammary approach, where direct visualization of the lesion from the posterior surface of the breast eliminates the need to triangulate its location based on skin markings or localizing wires entering the anterior surface of the breast. Intraoperative ultrasound, used alone or in conjunction with wire localization, can also improve the width of surgical margins and minimize the removal of excessive breast tissue [18].

### 4.5. Consider Breast MRI to Evaluated Disease Extent

There is considerable ongoing controversy regarding the value for contrast-enhanced breast MRI in the preoperative planning of breast cancer surgery [19, 20]. Although there is growing evidence that MRI may not alter the re-excision rate after breast conserving surgery, the breast reshaping or remodeling that occurs in oncoplastic breast surgery makes it imperative to obtain clear surgical margins at the initial operation. For this reason, there may be greater rationale for performing preoperative contrast-enhanced breast MRI in oncoplastic surgery [21]. Nevertheless, the sensitivity of contrast-enhanced MRI and the potential for false positive findings necessitate histological confirmation of MRI findings (ideally with ultrasound or MRI-guided core needle biopsy) before significantly altering the oncoplastic approach or converting to mastectomy.

### 4.6. Using a Surgical Drain

Contrary to the lumpectomy patient where seroma formation transiently preserves breast contour, the goal of oncoplastic surgery is to provide durable volume reconstruction by redistributing the breast parenchyma. Since large potential spaces are created in performance of oncoplastic surgery, a surgical drain may be considered to prevent accumulation of a large seroma that might complicate wound healing and recovery by exerting excessive tension on the breast and incision.

### 4.7. Orient the Surgical Specimen

Accurate orientation of the surgical specimen is essential to ensuring quality of breast cancer care. Nowhere is this more important than in oncoplastic surgery, where failure to accurately orient the surgical specimen may necessitate wide re-excision of close or positive margins, compromising the cosmetic outcome and possibly requiring conversion to mastectomy [22].

### 4.8. Obtain Intraoperative Pathology Consultation

The importance of attaining clear surgical margins at the initial resection cannot be overemphasized in oncoplastic surgery. To lower the risk of oncologic failure, surgeons should liberally utilize intraoperative pathology consultation including gross and frozen section analysis of surgical margins and specimen radiography [23]. If margin re-excision is required, re-excision of the entire affected margin should be performed to ensure that the new final margin mirrors the entire original surgical margin.

### 4.9. Preserve Sensation of the Nipple-Areolar Complex

The lateral cutaneous branch of the fourth intercostal nerve is the predominant source of sensory innervation for the nipple-areolar complex and maintains a relatively constant course through the breast [24]. In the left breast, the nerve generally exits the chest wall at the lateral border of the pectoralis minor, enters the posterior-lateral surface of breast at approximately the 4 o'clock position, and then traverses the glandular tissue to the inner areola along the 5 o'clock axis. In the right breast, the nerve generally enters the posterolateral surface of the breast at the 8 o'clock position and traverses the gland to enter the right areola along the 7 o'clock axis. Avoiding the trajectory of these nerves, particularly when performing circumareolar incisions and advancement flaps, will preserve nipple-areolar complex sensation and improve the quality of life of breast cancer patients.

### 4.10. Obtain Preoperative and Postoperative Photos

Documentation of the preoperative appearance and postoperative results of oncoplastic surgery will help the surgeon evaluate and improve his or her results over time. In addition, the confidential sharing of these photos with prospective patients will give them a clearer understanding of what they may expect from oncoplastic surgery.

## 5. Patient Selection

**Figure 1:**
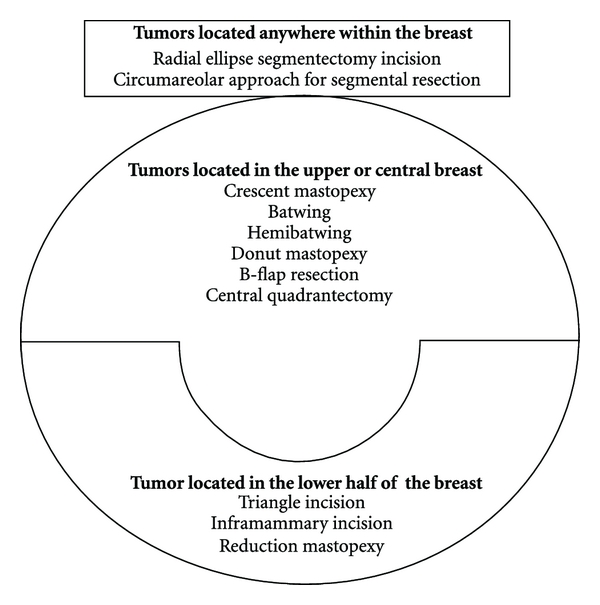
List of oncoplastic breast conserving procedures discussed in this paper, organized by tumor location.

Oncoplastic surgical procedures may be used for a wide range of breast cancer patients to achieve resection of breast cancers with acceptable and improved breast appearance. Figure 1 shows a list of selected oncoplastic approaches to BCS that may be performed by general surgeons and breast surgeons with appropriate training. Choosing the best operation for a given patient depends upon her tumor features, breast size and shape, and the surgeon's skill. Omitted from this paper are the oncoplastic breast surgery procedures that involve the use of a myocutaneous tissue flap (e.g., latissimus dorsi miniflaps) since these procedures will likely remain beyond the skill set of most general and breast surgeons.

## 6. The Advancement Flap

The advancement flap or adjacent tissue transfer is a fundamental technique common to all volume displacement oncoplastic procedures and should be mastered by any surgeon seeking to incorporate oncoplastic surgery in his or her surgical practice. The advancement flap is performed by dividing the retromammary fat plane posterior to the breast at the level of the muscular fascia to allow mobilization and displacement of the breast gland relative to the underlying pectoralis major muscle and chest wall. This technique is most easily practiced in conjunction with the segmentectomy incision, in which skin, parenchyma, and pectoralis fascia surrounding a cancer are excised en bloc, followed by mobilization and redistribution of the adjacent dermoglandular tissue to obliterate the resulting surgical cavity. In dissecting the retromammary fat plane, care should be taken to preserve most of the medial and lateral perforator blood vessels that are important to supporting the dermoglandular tissue flaps. While the breast's redundant blood supply generally lends itself to wide mobilization and undermining, injudicious division of perforating vessels may devascularize glandular or dermoglandular flaps, causing partial or complete flap loss. Extensive mobilization of fatty or elderly breasts may also increase the risk of fat necrosis.

## 7. Tumors Located Anywhere within the Breast

### 7.1. Radial Ellipse Segmentectomy

**Figure 2:**
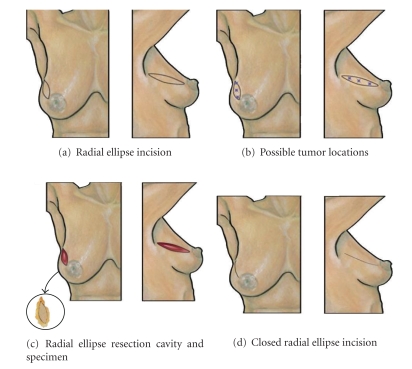
Radial ellipse segmentectomy. (a) Shows location of radial ellipse segmentectomy skin incision in upper outer quadrant. (b) Shows multiple “stars” indicating possible tumor locations suitable for this approach. (c) Shows resection cavity following excision of malignancy with excised specimen (inset). (d) Shows breast following closure of the skin incision.

The *radial ellipse segmentectomy* is a versatile procedure that can be used to resect a breast cancer located in any quadrant of the breast [12, 25, 26] (Figure 2). The specimen consists of an ellipse of skin, glandular tissue, and the underlying pectoralis fascia and is an ideal approach for resection of lesions lying adjacent to the skin or chest wall or extending radially toward the nipple. The width of the incision is designed to provide sufficient anterior margin clearance for a superficial lesion. For deep lesions, a narrower skin margin reduces skin removal. As an elliptical incision, the length of the ellipse is generally 3 times the width. The width and length of the glandular component generally approximate the dimensions of the skin margin, with emphasis placed on maintaining a macroscopic glandular margin of 1 cm or more to maximize the potential for microscopically clear margins. Attention should be paid to maintaining a glandular dissection plane relatively perpendicular to the skin surface to avoid unintended widening or narrowing of the specimen as the dissection extends posteriorly through the gland. Avoiding excessive retraction of the highly mobile breast gland can prevent inadvertent tangential dissection through the glandular tissue.

To prepare for wound closure, full-thickness dermoglandular advancement flaps are created by undermining the gland perpendicular to the long axis of the segmentectomy cavity. The degree of undermining depends upon the width of the surgical cavity and should be assessed intermittently by briefly approximating the surgical margins to determine if full-thickness wound closure can be accomplished without excessive tension. Minimal undermining is needed at the two apices of the elliptical cavity since these areas may not require mobilization for wound closure. Full-thickness wound closure is initiated by approximating the long axis of the surgical margins using 2–0 or 3–0 interrupted absorbable sutures placed at the posterior aspect of the glandular tissue, followed by placement of 1 or 2 additional suture layers to close the middle and anterior depth of the glandular tissue. If glandular dissection extended posterior to the nipple-areolar complex, special attention should be given to achieving an adequate full-thickness closure of the central and retroareolar tissue to prevent nipple-areolar complex retraction into an underlying cavity. Wound closure is completed by approximation of the skin in one or two layers using small gauge absorbable sutures.

The oncological advantages of the radial ellipse segmentectomy and advancement flap should be readily apparent: resection of skin makes a close or positive superficial margin irrelevant oncologically and excision of the pectoralis fascia eliminates the need for re-excision of a close or positive deep margin. The obvious disadvantages of this surgical approach (i.e., removal of proportionally more breast tissue near the apices of the ellipse resection and the need for longer incisions) are largely overcome by the advantage of reconstructing the breast mound and expanding the options of breast conserving surgery for patients who may be unsuitable candidates for the standard lumpectomy incision. This approach also minimizes the breast deformity commonly produced by resection of tumors from the central, medial, and inferior quadrants. Use of the radial ellipse segmentectomy is generally discouraged for resection of upper inner quadrant lesions where the resulting scar would be visible in the cleavage or above the bra.

### 7.2. Circumareolar Approach for Segmental Resection

**Figure 3:**
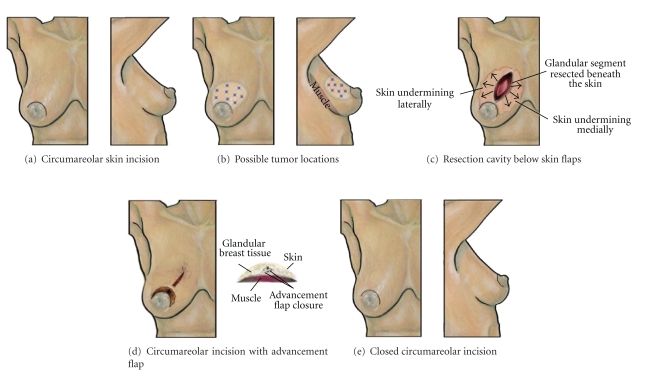
Circumareolar approach for segmental resection. (a) Shows location of circumareolar skin incision. (b) Shows multiple “stars” indicating possible tumor locations suitable for this approach. (c) Shows lumpectomy cavity after segmental resection of breast glandular tissue only with arrows denoting the extent of undermining of the overlying skin flap. (d) Shows results of glandular flaps advancements that allow the medial and lateral margins to be sutured together below the skin flap. Frontal and transverse views are shown. (e) Shows breast following closure of the circumareolar incision.

The *circumareolar approach for segmental resection* is a useful alternative to the radial ellipse segmentectomy when a radially oriented scar is undesired, such as in the upper inner quadrant of the breast (Figure 3). However, this versatile approach may be performed in any quadrant of the breast. Since the circumareolar approach fully preserves the skin overlying the lesion, this method should be restricted to resection of breast cancers that do not approximate the skin to minimize the risk of a positive superficial margin. With skin preservation, the surgical specimen consists of an elliptical or wedge-shaped mass of glandular tissue and the underlying pectoralis fascia. Placement of localizing wires superficial to, as well as on either side of, a nonpalpable lesion will improve margin clearance. 

Before beginning the procedure, the location of the lesion is marked on the overlying skin as a reference and the adjacent areolar margin is outlined to indicate the incision placement. A circumareolar incision extending up to 1/3 the circumference of the areola usually provides sufficient access for tumor resection of smaller tumors in patients with medium to large size areolas. Patients with small areolas or larger tumors are best managed using the *donut mastopexy *(*round block*)* resection technique*, which allows greater access to, and mobilization of, the breast gland [7].

The circumareolar approach for segmental resection is initiated by incising the areolar margin to enter the subcutaneous plane, which is then dissected widely over the quadrant of the breast containing the malignancy to create sufficient space for resection of the tumor with adequate margins. In general, the skin flap should extend from the areolar margin to the periphery of the breast and span a minimum of 25% of the breast surface area. Wider skin flap dissection is needed for larger breast resections. The surgeon should obtain an adequate superficial skin margin overlying the malignancy while also maintaining sufficient subcutaneous fat under the skin flap to ensure adequate skin perfusion. Placement of localizing wires superficial to the lesion will improve superficial margin clearance. An elliptical or wedge-shaped incision is then made in the breast parenchyma to encompass the breast malignancy and a gross margin of 1 cm or more. Localizing wires, if used, may be redirected below the skin flap to aid tumor resection. The parenchymal dissection is continued posteriorly until the underlying muscular fascia is encountered, and then dissection is extended posterior to the malignancy to remove the muscular fascia in continuity with the specimen. 

Wound closure is accomplished by undermining and performing an advancement flap of the breast gland in a direction perpendicular to the long axis of the segmentectomy cavity. The extent of dissection of the retromammary fat plane should be sufficient to allow tension-free approximation of the surgical margins. Since only the glandular flaps are advanced independent of the overlying skin, widening of the skin flaps may be necessary to prevent skin tethering and to allow free and independent movement of the glandular flaps. The procedure is completed with a layered closure of the glandular tissue using loosely applied 2–0 or 3–0 interrupted absorbable sutures as to avoid tissue strangulation and necrosis. Layered skin closure is completed using smaller gauge absorbable sutures.

## 8. Tumors Located in the Upper or Central Breast

### 8.1. Crescent Mastopexy Resection

**Figure 4:**
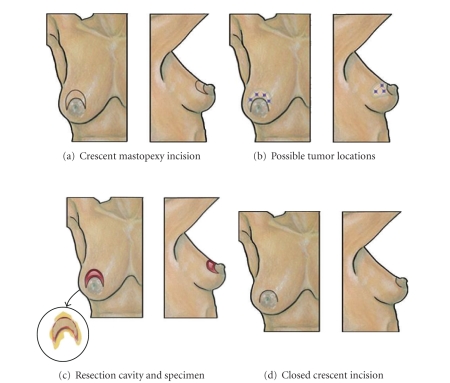
Crescent Mastopexy. (a) Shows location of crescent mastopexy skin incision. (b) Shows multiple “stars” indicating possible tumor locations suitable for this approach. (c) Shows resection cavity following excision of malignancy with excised specimen (inset). (d) Shows breast following closure of the skin incision.

The *crescent mastopexy resection* allows removal of a cancer in the central breast superior to but not involving the nipple or areola (Figure 4) [27]. The ideal lesion location for the crescent mastopexy resection is the periareolar 10 to 1 o'clock position. Use of this procedure for more medial or lateral lesions will displace the nipple-areolar complex in a direction that is generally considered undesirable. The crescent mastopexy resection consists of a crescent-shaped area of skin and glandular tissue excised from the superior border of the areola, which has the effect of elevating the nipple-areolar complex and inferior breast and achieving mild correction of ptosis. As an alternative to the standard circumareolar incision, the principle oncological advantage of the crescent mastopexy resection is the removal of skin overlying a tumor in the superficial breast, thus ensuring a clear superficial margin. 

The crescent mastopexy incision is designed by drawing two semiparallel “C-” shaped lines superior and adjacent to the areola, encompassing the skin immediately overlying a breast malignancy. The technical limitation to the crescent-shaped incision is the significant skin length disparity between the upper (longer) and lower (shorter) skin margins, which can be partly overcome during closure of the skin incision by taking larger horizontal suture bites along the longer skin margin and shorter vertical sutures bites along the shorter margin. This produces an areola with a slightly larger diameter. In general, breasts with larger areolas or smaller lesions size will be more accommodating of the crescent mastopexy approach. Smaller areolas or larger lesions may necessitate the use of the *batwing *or* hemibatwing resections*. 

The procedure is performed with the breast centrally positioned on the pectoralis muscle. The skin and glandular tissue surrounding the breast malignancy are incised to resect the lesion and a wide gross margin. While extension of the dissection to the pectoralis muscle will facilitate wound closure for smaller breasts and larger lesions, dissection to the muscle is generally unnecessary in larger breasts where sufficient central breast volume allows simple approximation of the superior and inferior glandular margins. When small breast size or large lesion size call for full-thickness dermoglandular and pectoralis fascial resection, wound closure is accomplished by undermining the adjacent glandular tissue in the retromammary fat plane and advancement of the superior and inferior dermoglandular margins to permit layered closure of the glandular tissue and skin.

### 8.2. Batwing Resection

**Figure 5:**
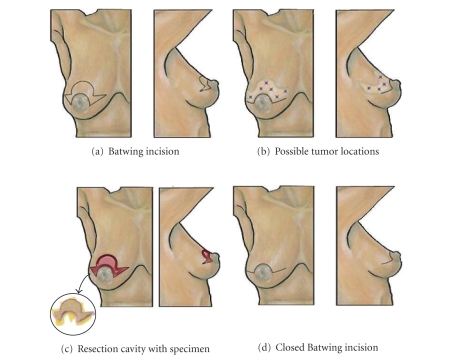
Batwing resection. (a) Shows location of batwing skin incision. (b) Shows multiple “stars” indicating possible tumor locations suitable for this approach. (c) Shows resection cavity of batwing resection with excised specimen (inset). (d) Shows breast following closure of the hemibatwing incision.

The *batwing resection* may be used as an alternative to the *crescent mastopexy resection* for wide excision of a breast malignancy located in the upper central aspect of the breast within a few centimeters of, but not directly involving, the nipple (Figure 5) [12, 27]. The batwing resection consists of a crescent-shaped central area of skin and gland adjoining 2 triangle-shape or wing-like areas of skin and gland extending from both sides of the areola. Similar to the crescent mastopexy resection, the batwing incision permits correction of breast ptosis by elevating the lower half of the breast and nipple-areolar complex. However, since the skin and glandular incision extends both medially and laterally to the nipple-areolar complex, the batwing incision also permits resection of a larger lesion that extends a few centimeters medial and/or lateral to the nipple-areolar complex. In addition, the large area of skin and glandular tissue that may be resected with the batwing resection allows for greater correction of ptosis than is possible with the crescent mastopexy resection. The cosmetic result is a smaller, less ptotic breast possessing two horizontal scars (at the 9-10 o'clock and 2-3 o'clock positions) connected by a less visible circumareolar incision at the upper half of the areola. A mastopexy of the opposite breast will correct breast asymmetry.

To perform the batwing resection, a batwing-shaped incision is drawn on the skin to encompass the skin overlying the breast malignancy. The lower half of the drawing should extend along the upper half of the areolar margin. The upper central edge of the batwing incision will ultimately become the new superior areolar margin. To prevent excessive lateral displacement of the nipple-areolar complex, the nipple should remain centered on a line extending from the native nipple location to the junction of the inner and middle thirds of the clavicle (approximately 8–10 cm from mid-sternal notch). This will move the nipple-areolar complex slightly more medial as it is moved to a higher position in the breast. 

The batwing incision is performed with the breast positioned centrally on the pectoralis muscle. After planning the incisions, the skin and glandular tissue are incised and dissection is carried out posterior to the breast malignancy. Depending on the position of the lesion, the surgeon may bias the glandular resection in one direction or the other to gain greater clearance around the malignancy and to preserve glandular tissue where it may be advantageous to do so. For wound closure, the glandular tissues cranial and caudal to the resection cavity are advanced together to permit layered closure of the glandular tissue and skin with absorbable sutures.

### 8.3. Hemibatwing Resection

**Figure 6:**
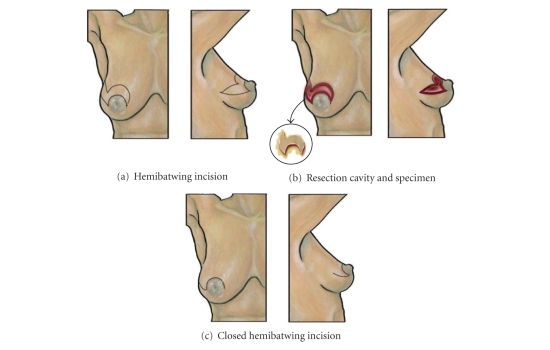
Hemibatwing resection. (a) Shows location of hemibatwing skin incision. (b) Shows resection cavity of hemibatwing resection with excised specimen (inset). (c) Shows breast following closure of the hemibatwing incision.

As its name suggests, the *hemibatwing resection* is similar to the *batwing resection* except that only one “wing” is excised (Figure 6) [27]. The optimal use of the hemibatwing resection is wide local excision of an upper outer quadrant periareolar lesion that extends along the 9-10 o'clock (right) or 2-3 o'clock (left) axis, where removal of skin, glandular tissue, and pectoralis fascia can optimize the surgical margins and provide mild correction of ptosis. Hemibatwing resections are less commonly used for medial quadrant lesions where an incision extending into the upper inner quadrant would leave a visible scar in the cleavage. Aside from these important distinctions, the hemibatwing resection is performed in a manner essentially identical to the batwing resection.

### 8.4. Donut Mastopexy Resection

**Figure 7:**
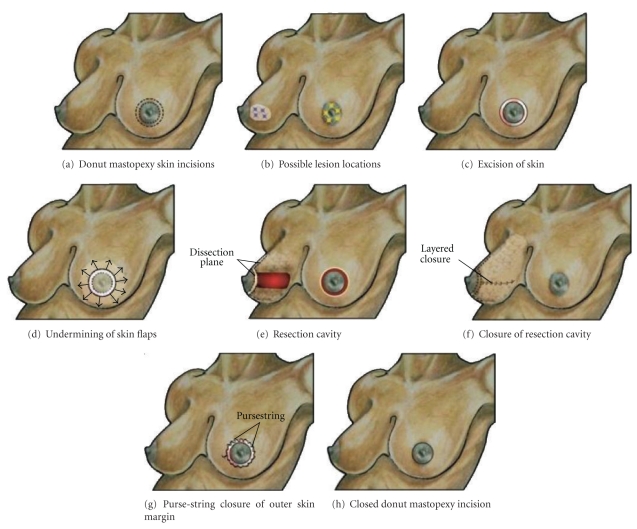
Donut mastopexy resection. (a) Shows location of two circumareolar incisions. (b) Shows frontal and profile views of the breasts with multiple “stars” indicating possible tumor locations suitable for this approach. (c) Shows the area of de-epithelized or excised skin at edge of areola. (d) Shows arrows denoting undermining of skin flaps in the central breast. For illustration purpose, (e) shows medial profile view of the right breast with central lumpectomy cavity and area of undermined skin flaps. Frontal view of left breast shows central lumpectomy behind nipple-areolar complex. (f) Shows results of advancement of glandular tissue which is mobilized and sutured together to fill the central breast. (g) Shows reduction of the diameter of the outer skin margin using a purse-string suture. (h) Shows breast following closure of the skin incision.

The *donut mastopexy* or* round block technique* (Figure 7) allows generous access to any quadrant of the breast while confining the incision to the areolar margins [7, 12, 28, 29]. Similar to the nipple-areolar sparing mastectomy, the donut mastopexy technique is best utilized in the setting of a malignancy that does not extend to the skin or nipple-areolar complex. The donut mastopexy utilizes a pair of concentric circumareolar skin incisions; one placed at the areolar margin and a second whose radius is at least 1 cm longer. The intervening ring of skin is excised (either full thickness or partial thickness) and wide skin flaps are developed over the index and flanking quadrants to enable wide local excision of the malignancy and the adjoining pectoralis fascia. Placement of localizing wires anterior and adjacent to the malignancy will improve margin clearance. Following tumor resection, reconstruction of the gland is undertaken by undermining, advancing, and performing a layered closure of the flanking glandular breast tissue using 2–0 absorbable sutures. If full-thickness, full-circumferential, skin incisions are utilized, special attention must be taken to minimize the undermining of the nipple-areolar complex which would compromise the blood supply from the underlying glandular tissue. For skin closure, an absorbable purse-string suture is placed in the outer skin margin to reduce its diameter to that of the normal areola. Skin closure is then completed with the suturing of these two skin margins together, forming the new areolar margin.

A primary advantage of the donut mastopexy resection and the reduction of the skin envelope is the lifting effect that it has on the breast. Cosmesis can further be enhanced by the asymmetric, more cephalad placement of the larger concentric circle, which produces further elevation of the nipple-areolar complex upon wound closure. The principle technical disadvantages of this approach are its greater complexity and the nipple-areolar complex denervation that results from full-thickness circumferential incision of the areolar margin.

### 8.5. B-Flap Resection

**Figure 8:**
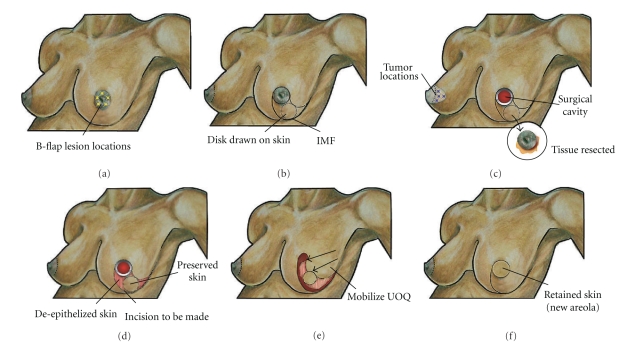
B-flap resection. (a) Shows multiple stars with possible tumor locations. (b) Shows location of skin incision, including disk of skin to be preserved. (c) Shows surgical cavity after excision of central lumpectomy with removal of nipple-areolar complex (inset). For illustration purposes, medial profile view of right breast shows multiple “stars” indicating possible tumor locations suitable for this approach. (d) Shows surgical cavity, areas of de-epithelized skin, preserved disk of epithelized skin, and location of incision to be made in the glandular tissue. (e) Shows advancement and clockwise rotation of lower outer quadrant until disk of skin occupies the nipple-areolar complex position. (f) Shows breast following approximation and closure of the skin incisions. De-epithelized skin is buried below the skin of the lower inner quadrant and the disk of skin forms a new areola.

When proximity of the tumor to the nipple-areolar complex necessitates resection of the nipple-areolar complex, the* B-flap resection* (the Grisotti mastopexy technique) is the ideal approach for reconstructing the central breast in a woman with sufficient breast volume or moderate breast ptosis (i.e., ≥8 cm distance from the nipple to the inframammary skin fold) (Figure 8) [30–33]. The B-flap resection is named for the “B-” shaped incision that is created to resect and reconstruct the breast. The circumareolar incision makes up the upper portion of the “B” and the lower portion of the “B” is defined by a disk of skin from the lower part of the breast that is preserved and transposed (along with an inferior pedicle of glandular tissue) to the central breast to replace the resected areola and reconstruct the central breast defect. The resulting surgical specimen is comprised of the nipple-areolar complex and the central cylinder of glandular tissue extending to the pectoralis fascia. 

In designing the B-flap resection, the breast is positioned centrally on the pectoralis muscle and the areolar margin is outlined. The diameter of the areola is measured and then a disc of skin of equal diameter is drawn on the skin of the breast just inferior to the nipple-areolar complex. This disc of skin will form the new areola. For an eccentrically placed tumor, a larger circumareolar incision can be designed to encompass the skin anterior to the lesion. In this instance, the diameter of the disc of skin should be based on the diameter of the normal areola to maintain symmetry. On the other hand, use of a larger circumareolar incision allows flexibility in positioning the nipple-areolar complex to correct ptosis. Next, two curvilinear lines are drawn from the lateral and medial edges of the native areola and skin disc. As the lines pass inferior to the skin disc, both are curved inferolaterally to converge at the lateral aspect of the inframammary skin fold. 

B-flap resection begins with incision of the areolar margin. Dissection is continued posteriorly while maintaining a generous gross margin around the malignancy until the pectoralis muscle is encountered. After resecting the specimen, the area of skin outlined in the inferolateral breast is de-epithelialized except for the encircled disc of skin that will form the future areola. De-epithelization is easily accomplished using tenotomy scissors to excise the pigmented epithelial layer, leaving intact the white reticular “deep” dermis layer. 

Wound closure is initiated by incising the breast and inframammary fold along the medial edge of the de-epithelialized skin, extending this incision through the underlying glandular tissue to the chest wall. The lower outer quadrant of the breast is then dissected off the underlying chest wall to allow superior and medial rotation and advancement of the skin disc and underlying glandular tissue to the central breast. This brings the skin disc to the position of the original nipple-areolar complex and partially restores the volume of the central breast using glandular tissue from the inferior and outer quadrants. The central breast mound is further reconstituted by suturing the transposed tissue to the surgical margin using interrupted 2–0 or 3–0 absorbable sutures in multiple layers. Partial undermining of the lower inner quadrant facilitates full-thickness approximation of the lower breast gland. Layered closure of the skin is then performed by suturing the edge of the skin disk to the original areolar skin margin and approximating the epithelialized skin margins of the infero-lateral breast after burying the de-epithelialized dermis below the skin surface. Reconstruction of the nipple and tattooing of the areola may be completed at a later date.

### 8.6. Central Quadrantectomy

**Figure 9:**
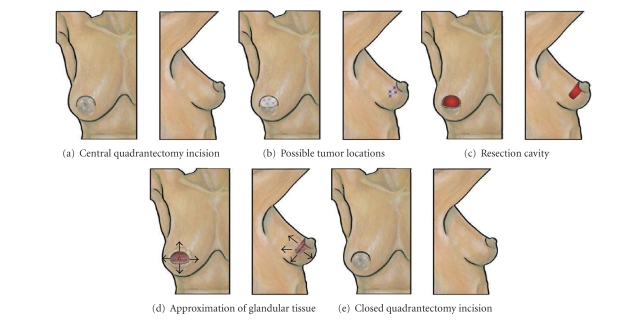
Central quadrantectomy. (a) Shows location of central quadrantectomy skin incision. (b) Shows multiple “stars” indicating possible tumor locations suitable for this approach. (c) Shows resection cavity following excision of malignancy. The nipple-areolar complex was omitted in the left image to allow visualization of surgical cavity. (d) Shows results of glandular flap advancements that allow the surgical margins to be sutured together using purse-string sutures to obliterate the surgical cavity. Arrows showing undermining of skin flaps in the central breast. Nipple-areolar complex omitted in the left image to allow visualization of the approximated glandular tissue. (e) Shows breast following closure of the skin incision.

*Central quadrantectomy* is performed through a circumareolar incision spanning up to 50% of the areolar circumference and may be used in patients with widely ranging breast sizes [30, 31] (Figure 9). The surgical specimen consists of a cylinder of breast tissue extending from the subareolar plane to the pectoralis muscle encompassing the breast malignancy with a generous surgical margin. Localizing wires inserted along the anterior medial and anterior lateral aspects of the malignancy will facilitate dissection of the subareolar tissue plane and optimize clearance of the surgical margins. When proximity of tumor to the nipple requires resection of the nipple-areolar complex, the nipple-areolar complex is removed in continuity with the cylinder of underlying glandular tissue. 

Beginning with the circumareolar skin incision, the dissection is carried out subareolarly to create a dermoglandular flap of the nipple-areolar complex. Dissection of this plane is then extended peripherally in all directions for several centimeters to separate the central breast skin from the central breast gland. This will facilitate resection of the specimen and subsequent wound closure. Maintaining a relatively thick areolar skin flap is important to ensuring adequate perfusion. Once the areolar flap and adjacent skin are detached from the central breast mound, the localizing wires bracketing the lesion are then identified in the subcutaneous plane and used to define the gross margins of the central breast resection. Dissection of the central cylinder is then extended to the pectoralis muscle from which it is detached along with the muscular fascia. 

Wound closure is initiated by placement of 2-3 layers of purse-string sutures at the posterior, middle, and anterior depths of the cylindrical resection cavity to bring the central breast mound together in the retroareolar position. Additional undermining of the skin flaps may be needed to release areas of skin that become tethered when the central gland is approximated. Closure of the areolar margin is completed by reapproximation and layered closure of the subcutaneous tissue and skin.

## 9. Tumor Located in the Lower Half of the Breast

### 9.1. Triangle Resection

**Figure 10:**
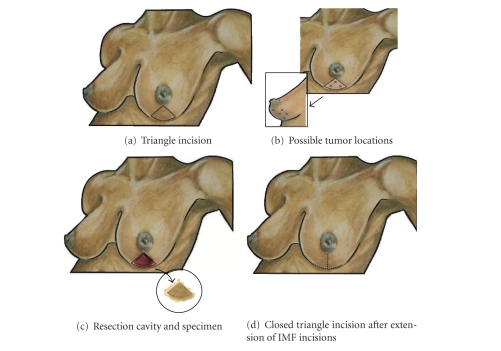
Triangle resection. (a) Shows location of the triangular skin incision. (b) Shows multiple “stars” indicating possible tumor locations suitable for this approach. (c) Shows resection cavity following excision of malignancy with excised specimen (inset). (d) Shows breast following closure of the skin incision, including extensions of incision along the inframammary skin fold.

Resection of a breast malignancy from the lower half of the breast has significant potential to cause breast disfigurement. The standard lumpectomy performed in this location may produce a “bird beak” deformity in which the nipple-areolar complex or central breast overhangs a concave area in the inferior breast. Such disfigurement can be avoided by the use of the triangle resection which is capable of achieving wide excision of lesions in the 5–7 o'clock region of the breast while allowing reconstitution of the inferior pole of the breast by advancement of adjacent tissues into the surgical cavity (Figure 10). The triangle incision is ideally suited for lesions that are radially oriented or approximate the skin, but it is also useful for deeper lesions. Using this technique, the resulting full-thickness wedge-shaped specimen of skin and glandular tissue is allows removal of a relatively large lesion in this location [27]. 

To perform the triangle resection, a triangular or wedge-shaped incision is drawn on the skin overlying the breast lesion. The base of the triangle should intersect the inframammary skin fold and the apex of the triangle should point toward, but not necessarily extend to, the inferior areolar margin. Dissection is begun by incising the triangular area of skin and dividing the underlying glandular tissue down to the chest wall. Resection of the specimen is completed by extending the plane of dissection posterior to the specimen at the surface of the serratus anterior muscle or rectus fascia down to the inframammary fold, which is subsequently divided. If necessary, the rectus fascia and/or serratus anterior may be resected to ensure a negative deep margin posterior to the specimen. Special attention should be paid to maintaining a glandular dissection plane that is relatively perpendicular to the skin to facilitate approximation of the surgical margins. Caution should also be taken to avoid excessive traction of the specimen during the course of dissection, as this may lead to inadvertent dissection behind and cephalad to the nipple and removal of excessive normal glandular tissue.

For wound closure, the adjacent lower outer and lower inner quadrants must be brought together to allow full-thickness approximation of the glandular tissue. This is accomplished by extending the inframammary fold incision toward the medial and lateral edges of the breast, undermining the lower half of the breast to create lower outer quadrant and lower inner quadrant dermoglandular flaps, and approximating the dermoglandular flaps using multiple layers of 2–0 or 3–0 absorbable sutures. The resulting length discrepancy between the breast and inframammary fold skin edges can generally be easily overcome by temporary approximation of the edges with skin staples, redistribution of the shorter edge along the longer edge, and use of suturing techniques described above (see Section 8.1). To avoid excessive tension on the breast skin edges, the inframammary fold incision may be extended medially and laterally to allow additional mobilization of the dermoglandular flaps. To complete the procedure, the inframammary fold is closed in multiple layers by approximating the glandular and fibrous tissue of the breast with the fibrous tissue of the inframammary fold using 2–0 or 3–0 interrupted absorbable sutures, followed by closure of the skin with a smaller gauge suture. 

From a perfusion perspective, the most vulnerable parts of the breast dermoglandular flaps are the distal corners of the medial and lateral flaps where they converge at the inframammary fold [27]. Limited collateral blood flow at these corners makes them susceptible to ischemia, leading to partial- or full-thickness necrosis of the corners. This can be minimized with delicate tissue handling of the corners, minimizing tension in wound closure, and avoiding the use of retracting instruments on these corners which have the tendency to further traumatize the skin and underlying breast tissue. An additional strategy is to “round off” these corners to reduce the risk of underperfusion of the distal corners. The resulting “skin defect” can be filled by preserving a comparable area of skin at the midpoint of the inframammary fold.

### 9.2. Inframammary Resection

**Figure 11:**
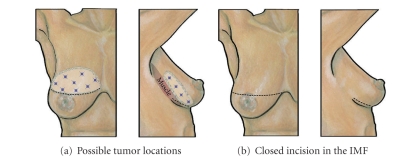
Inframammary Resection. (a) Shows location of inframammary skin incision. “Stars” indicate multiple possible tumor locations suitable for this approach. (b) Shows breast following closure of the inframammary incision.

The *inframammary resection* is a versatile incision for removal of cancers from a variety of locations in the lower or posterior aspects of the breast [27] (Figure 11). Resection of a breast malignancy via the inframammary approach places the incision in the inframammary skin fold where it is “hidden” behind and below the breast. Since the skin overlying the lesion is fully preserved, the inframammary approach should be restricted to resection of breast cancers that are not located in the superficial breast to minimize the risk of a positive superficial margin. Given the indirect or “back-door” approach of this resection, it is imperative that surgeon use multiple bracketing wires and/or intraoperative ultrasound when appropriate to ensure wide excision of the malignancy.

Using the inframammary approach, an incision is made in the skin of inframammary fold and extended through the subcutaneous and fibrous layers to the chest wall. The length of the incision depends, in part, on the size of the lesion, location of the lesion, and the degree of mobilization required to access the lesion and to close the surgical cavity. Larger and more cephalad lesions will require a longer incision to facilitate access to the upper breast. Smaller and more caudal lesions may be accessed through a shorter incision in the medial, central, or lateral inframammary skin fold. Dissection is then extended through the retromammary fat plane to a position at least 3 cm cephalad to the malignancy, the position of which is determined using bimanual palpation, skin markings, localizing wires, ultrasound, or some combination of these techniques. An incision is then made in the posterior surface of breast in the perimeter of the lesion and then extended anteriorly to widely resect the tumor with a generous superficial margin. Placement of localizing wires superficial to the lesion will improve superficial margin clearance. If localizing wires are used, the localizing wires should be identified within the substance of the gland and the external ends of the wires should be redirected so that they project out of the posterior surface of the breast into the surgical cavity. With the localizing wires in view, resection of the specimen is carried out by widely excising the localizing wires and the bracketed specimen. Resection of the corresponding area of muscular fascia should also be considered for deeper lesions.

Wound closure is initiated by approximating the surgical margins to prevent or minimize retraction of the skin into the underlying surgical cavity. This step can be performed relatively easily since the breast has already been widely mobilized from the chest wall. However, if additional mobilization is needed, dissection of the retromammary fat plane or subcutaneous tissue plane can be carried out to facilitate cavity closure. Final wound closure is completed by reapproximation of the inframammary using 2–0 or 3–0 absorbable sutures followed by layered closure with smaller gauge absorbable sutures.

### 9.3. Reduction Mammaplasty

The *reduction mammaplasty resection* combines wide local excision of a breast malignancy with reduction mammaplasty in a patient who desires breast reduction. Reduction mammaplasty resection may be performed with or without nipple preservation depending on the location of the cancer. When the nipple-areolar complex is preserved, recentralization of the nipple is generally performed to move the nipple-areolar complex to a more anterior and superior position on the breast mound. The principal oncological advantage of the reduction mammaplasty is the ability to achieve wide local excision of large breast malignancies, especially those that might not be amendable to breast conserving surgery using the standard lumpectomy. Breast rearrangement is useful for masking larger segmental defects and simultaneously creating an aesthetic breast mound. The versatility of this approach makes it suitable for resection of lesions located between the 4–8 o'clock axes of the breast, as well as in the retroareolar or supra-areolar position. The reduction mammaplasty resection is regarded to be among the more complex oncoplastic breast conserving procedures and should not be performed by surgeons lacking appropriate training in plastic or oncoplastic surgery. Its inclusion in this paper is meant to provide a broad overview of the approach for surgeons considering appropriate training in oncoplastic surgery. 

**Figure 12:**
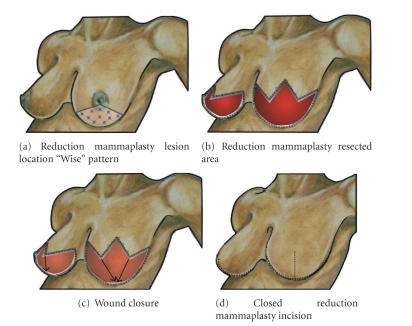
Reduction mammaplasty. (a) Shows multiple “stars” indicating possible tumor locations suitable for this approach. (b) Shows surgical cavity after resection of nipple-areolar complex and inferior breast using Wise pattern. A symmetrical reduction is shown in the opposite breast. (c) Shows advancement of medial and lateral pedicles to inframammary fold. (d) Shows the breasts after closure of the wounds.

The key foundations of any reduction technique include the preservation of the vascular supply to the nipple-areolar complex and vascular supply to the remaining breast parenchyma. The first technical aspect of the reduction mammaplasty resection is the planning of the skin incision. While the traditional approach has been the “Wise pattern” (keyhole) incision (Figure 12) [34, 35], vertical reduction techniques have become very popular as well [36]. The “Wise pattern” creates the classic inverted “T-” or anchor-shaped incision upon closure of the wound and is usually incorporated with an inferiorly based dermoglandular pedicle. When resection of the nipple-areolar complex is required, the central skin incision consists of an inverted “V”, the apex of which is placed just above the nipple-areolar complex. In general, the apex of the “V” is placed at the intersection of a longitudinal line extending from the junction of the inner and middle-third of the clavicle to the nipple (i.e., the breast meridian) and a second transverse line drawn at the level of the inframammary fold transposed onto the superior breast skin in the upright position. The apex of the “V” is usually 18–20 cm from the suprasternal notch. The point of intersection is the superior areolar point. From the superior areolar point, the two legs of the “V” should pass inferiorly to the left and right of the nipple-areolar complex for a length of 3–5 cm, plus an additional length of 5 cm or more for a total length of approximately 10 cm. From this point, lines are extended horizontally in the medial and lateral directions to join the medial and lateral ends of the inframammary fold. Skin marking is performed with the patient in the upright sitting or standing position [37].

When the position of the malignancy enables preservation of the nipple-areolar complex, the initial markings are drawn as described above, substituting an inverted “U-” shaped incision instead of a “V-” shaped incision with placement of the apex of the inverted “U” at the new superior areolar point. When the breast wound is closed, the vertical lines will span the distance from the inferior areolar point to the inframammary fold, and the horizontal lines will form the superior skin margin of the new inframammary fold. Modifications of the standard inferior, medial, or lateral incisions have also been described for breast conservation and include adjusting the incisions to incorporate the resected area [38]. 

After designing the skin incision, resection of the breast malignancy is undertaken by incising the inframammary fold, the affected skin, and the glandular margins down to the chest wall, maintaining the dissection plane at right angles to the skin surface. If nipple and areola preservation is intended, care should be taken to preserve vascular supply to the nipple-areolar complex either by avoiding undermining the nipple-areolar complex for a parenchyma pedicle or by maintaining the retained dermis along at least two-thirds of the areolar circumference. In addition, de-epithelialization of the skin between the remaining areolar margin and new superior skin margin may be used to optimize perfusion and innervation of the nipple-areolar complex. For lesions that are eccentrically located (e.g., in the 3-4 o'clock or 7-8 o'clock positions) the surgeon may chose to “cheat” the glandular resection medially or laterally to gain adequate clearance around the malignancy. After complete excision of the malignancy, dermoglandular flaps using the remaining breast tissue can also be incorporated to fill in significant defects [39]. 

Wound closure is initiated by tailor-tacking the previous incisions with staples, starting with the inferior, medial, and lateral incisions. If the originally drawn circular pattern at the apex of the Wise pattern (“U” shape) is symmetric, the nipple can also be inset, burying the de-epithelialized tissue under the incision. If better symmetry of the nipple-areolar complex is required, a cookie-cutter can be used to create a new superior incision margin after closure of the inverted “T” incisions only. Any additional skin removal is performed by de-epithelialization. When the nipple-areolar complex has been resected, the skin can be closed in either a transverse or vertical pattern, depending on the type of reduction performed. Layered closure of the parenchyma, dermis, and skin ensures maintenance of the final breast shape over time. 

Complications of combined reduction mammaplasty and malignancy excision occur in 17% of patients [39]. Skin and fat necrosis are the most common complications and occur more often in smokers and obese patients. Nipple-areola necrosis occurs in approximately 3% of patients.

## 10. Summary

The procedures presented herein constitute a broad overview of the most commonly performed oncoplastic breast conserving procedures for optimizing tumor resection and cosmesis. This overview provides a starting point for surgeons interested in adding oncoplasty to the surgical options that they offer to their patients. While some of the procedures can be introduced without specialized training, breast and general surgeons seeking advanced training in oncoplastic surgery should participate in a breast fellowship program or an oncoplastic surgery course. Oncoplastic surgery courses are offered by several specialty societies, including the American Society of Breast Surgeons and the American Society of Breast Diseases.

## References

[1] Abe O, Abe R, Asaishi K (1995). Effects of radiotherapy and surgery in early breast cancer: an overview of the randomized trials. *The New England Journal of Medicine*.

[2] Locker GY, Sainsbury JR, Cuzick J (2004). Breast surgery in the ’Arimidex, Tamoxifen Alone or in Combination’ (ATAC) trial: american women are more likely than women from the United Kingdom to undergo mastectomy. *Cancer*.

[3] Cochrane RA, Valasiadou P, Wilson ARM, Al-Ghazal SK, Macmillan RD (2003). Cosmesis and satisfaction after breast-conserving surgery correlates with the percentage of breast volume excised. *British Journal of Surgery*.

[4] Waljee JF, Hu ES, Newman LA, Alderman AK (2008). Predictors of breast asymmetry after breast-conserving operation for breast cancer. *Journal of the American College of Surgeons*.

[5] Rainsbury RM (2007). Surgery Insight: oncoplastic breast-conserving reconstruction—indications, benefits, choices and outcomes. *Nature Clinical Practice Oncology*.

[6] Noguchi M, Saito Y, Mizukami Y (1991). Breast deformity, its correction, and assessment of breast conserving surgery. *Breast Cancer Research and Treatment*.

[7] Masetti R, Pirulli PG, Magno S, Franceschini G, Chiesa F, Antinori A (2000). Oncoplastic techniques in the conservative surgical treatment of breast cancer. *Breast Cancer*.

[8] Petit JY, Rietjens M, Garusi C, Greuze M, Perry C (1998). Integration of plastic surgery in the course of breast-conserving surgery for cancer to improve cosmetic results and radicality of tumor excision. *Recent Results in Cancer Research*.

[9] Baildam AD (2008). Oncoplastic surgery for breast cancer. *British Journal of Surgery*.

[10] Munhoz AM, Aldrighi CM, Ferreira MC (2007). Paradigms in oncoplastic breast surgery: a careful assessment of the oncological need and esthetic objective. *The Breast Journal*.

[11] Clough KB, Lewis JS, Couturaud B, Fitoussi A, Nos C, Falcou MC (2003). Oncoplastic techniques allow extensive resections for breast-conserving therapy of breast carcinomas. *Annals of Surgery*.

[12] Anderson BO, Masetti R, Silverstein MJ (2005). Oncoplastic approaches to partial mastectomy: an overview of volume-displacement techniques. *The Lancet Oncology*.

[13] Krawczyk JJ, Engel B (1999). The importance of surgical clips for adequate tangential beam planning in breast conserving surgery and irradiation. *International Journal of Radiation Oncology Biology Physics*.

[14] Burkholder HC, Witherspoon LE, Burns RP, Horn JS, Biderman MD (2007). Breast surgery techniques: preoperative bracketing wire localization by surgeons. *American Surgeon*.

[15] Kirstein LJ, Rafferty E, Specht MC (2008). Outcomes of multiple wire localization for larger breast cancers: when can mastectomy be avoided?. *Journal of the American College of Surgeons*.

[16] Ngô C, Pollet AG, Laperrelle J (2007). Intraoperative ultrasound localization of nonpalpable breast cancers. *Annals of Surgical Oncology*.

[17] Harlow SP, Krag DN, Ames SE, Weaver DL (1999). Intraoperative ultrasound localization to guide surgical excision of nonpalpable breast carcinoma. *Journal of the American College of Surgeons*.

[18] Moore MM, Whitney LA, Cerilli L (2001). Intraoperative ultrasound is associated with clear lumpectomy margins for palpable infiltrating ductal breast cancer. *Annals of Surgery*.

[19] Morris EA (2010). Should we dispense with preoperative breast MRI?. *The Lancet*.

[20] Schell AM, Rosenkranz K, Lewis PJ (2009). Role of breast MRI in the preoperative evaluation of patients with newly diagnosed breast cancer. *American Journal of Roentgenology*.

[21] Lebovic GS, Anderson BO (2009). Oncoplastic breast surgery: current status and best candidates for treatment. *Current Breast Ccancer Reports*.

[22] Molina MA, Snell S, Franceschi D (2009). Breast specimen orientation. *Annals of Surgical Oncology*.

[23] Fleming FJ, Hill ADK, Mc Dermott EW, O’Doherty A, O’Higgins NJ, Quinn CM (2004). Intraoperative margin assessment and re-excision rate in breast conserving surgery. *European Journal of Surgical Oncology*.

[24] Farina MA, Newby BG, Alani HM (1980). Innervation of the nipple-areola complex. *Plastic and Reconstructive Surgery*.

[25] Veronesi U, Luini A, Galimberti V, Zurrida S (1994). Conservation approaches for the management of stage I/II carcinoma of the breast: Milan Cancer Institute trials. *World Journal of Surgery*.

[26] Senofsky GM, Gierson ED, Craig PH, Spear SL (1998). Local excision, lumpectomy, and quadrantectomy: surgical considerations. *Surgery of the Breast: Principles and Art*.

[27] Nahabedian MY (2009). *Oncoplastic Surgery of the Breast*.

[28] Amanti C, Moscaroli A, Lo Russo M (2002). Periareolar subcutaneous quadrantectomy: a new approach in breast cancer surgery. *Il Giornale di chirurgia*.

[29] Amanti C, Regolo L, Moscaroli A, Lo Russo M, Catracchia V (2003). Total periareolar approach in breast-conserving surgery. *Tumori*.

[30] Wagner E, Schrenk P, Huemer GM, Sir A, Schreiner M, Wayand W (2007). Central quadrantectomy with resection of the nipple-areola complex compared with mastectomy in patients with retroareolar breast cancer. *The Breast Journal*.

[31] Naguib SF (2006). Oncoplastic resection of retroareolar breast cancer: central quadrantectomy and reconstruction by local skin-glandular flap. *Journal of the Egyptian National Cancer Institute*.

[32] Regnault P (1974). Reduction mammaplasty by the “B” technique. *Plastic and Reconstructive Surgery*.

[33] Grisotti A (1994). Immediate reconstruction after partial mastectomy. *Operative Techniques in Plastic & Reconstructive Surgery*.

[34] Georgiade NG, Serafin D, Morris R, Georgiade G (1979). Reduction mammaplasty utilizing an inferior pedicle nipple-areolar flap. *Annals of Plastic Surgery*.

[35] WISE RJ (1956). A preliminary report on a method of planning the mammaplasty. *Plastic and Reconstructive Surgery*.

[36] Lejour M, Abboud M, Declety A (1990). Reduction des cicatrices de plastie mammaire de l’ancre courte a la vertucale. *Annales de Chirurgie Plastique Esthétique*.

[37] Mathes S (2006). *Plastic Surgery*.

[38] Hudson DA (2007). A modified excision for combined reduction mammoplasty and breast conservation therapy in the treatment of breast cancer. *Aesthetic Plastic Surgery*.

[39] Munhoz AM, Montag E, Arruda EG (2006). Critical analysis of reduction mammaplasty techniques in combination with conservative breast surgery for early breast cancer treatment. *Plastic and Reconstructive Surgery*.

